# DExD/H-Box Helicase 36 Signaling *via* Myeloid Differentiation Primary Response Gene 88 Contributes to NF-κB Activation to Type 2 Porcine Reproductive and Respiratory Syndrome Virus Infection

**DOI:** 10.3389/fimmu.2017.01365

**Published:** 2017-10-23

**Authors:** Huiyuan Jing, Yanrong Zhou, Liurong Fang, Zhen Ding, Dang Wang, Wenting Ke, Huanchun Chen, Shaobo Xiao

**Affiliations:** ^1^State Key Laboratory of Agricultural Microbiology, College of Veterinary Medicine, Huazhong Agricultural University, Wuhan, China; ^2^Key Laboratory of Preventive Veterinary Medicine in Hubei Province, The Cooperative Innovation Center for Sustainable Pig Production, Wuhan, China

**Keywords:** porcine reproductive and respiratory syndrome virus, DExD/H-box helicase 36, nucleocapsid protein, NF-κB, pro-inflammatory cytokine

## Abstract

DExD/H-box helicase 36 (DHX36) is known to be an ATP-dependent RNA helicase that unwinds the guanine-quadruplexes DNA or RNA, but emerging data suggest that it also functions as pattern recognition receptor in innate immunity. Porcine reproductive and respiratory syndrome virus (PRRSV) is an *Arterivirus* that has been devastating the swine industry worldwide. Interstitial pneumonia is considered to be one of the most obvious clinical signs of PRRSV infection, suggesting that the inflammatory response plays an important role in PRRSV pathogenesis. However, whether DHX36 is involved in PRRSV-induced inflammatory cytokine expression remains unclear. In this study, we found that PRRSV infection increased the expression of DHX36. Knockdown of DHX36 and its adaptor myeloid differentiation primary response gene 88 (MyD88) by small-interfering RNA in MARC-145 cells significantly reduced NF-κB activation and pro-inflammatory cytokine expression after PRRSV infection. Further investigation revealed that PRRSV nucleocapsid protein interacted with the N-terminal quadruplex binding domain of DHX36, which in turn augmented nucleocapsid protein-induced NF-κB activation. Taken together, our results suggest that DHX36–MyD88 has a relevant role in the recognition of PRRSV nucleocapsid protein and in the subsequent activation of pro-inflammatory NF-κB pathway.

## Introduction

Virus infection induces innate immune responses through the activation of pattern recognition receptors (PRRs), which detect pathogen-associated molecular patterns (PAMPs) and are crucial for protection against microbial invasion and maintaining homeostasis (1). Previous studies have suggested involvement of various DExD/H-box RNA helicases, for example, DExD/H-box helicase 58 (also known as RIG-I) in the initiation of innate immune responses (2, 3). Recently, the involvement of other DExD/H-box RNA helicases, for instance, DExD/H-box helicase 36 (DHX36), in innate immunity has also been extensively studied (2–4).

DExD/H-box helicase 36, also termed RNA helicase associated with AU-rich RNA element, was initially characterized as an ATP-dependent RNA helicase that demonstrates high affinity for quadruplex (G4) structures in DNA and RNA (5). DHX36 contains a core helicase domain responsible for ATP-binding/helicase activity and is flanked on either side by N- and C-terminal extensions (5). Later, DHX36 has been shown to function as a myeloid differentiation primary response gene 88 (MyD88)-dependent sensor for herpes simplex virus 1 in the cytosol of plasmacytoid dendritic cells, and a TIR-domain-containing adapter-inducing IFN-β (TRIF)-dependent sensor in response to influenza and reovirus infection in myeloid dendritic cells (2, 3). Whether DHX36 can recognize other virus-derived PAMPs except nucleic acids has not been elucidated to date.

Porcine reproductive and respiratory syndrome virus (PRRSV) is an enveloped, positive-stranded RNA virus, which belongs to the family of *Arteriviridae* that are grouped together with the Coronaviruses into the order *Nidovirales* (6–8). The positive-stranded RNA genome of PRRSV is approximately 15 kb in length and is packaged by the nucleocapsid (N) protein, one of the most abundant viral proteins during infection (9, 10). Several pro-inflammatory cytokines including IL-6, IL-8, and TNF-α are significantly elevated during PRRSV infection and correlate with the persistent infection and tissue pathology associated with PRRSV (11–13).

Although lipopolysaccharides treatment results in enhanced production of pro-inflammatory cytokines during PRRSV infection, the intracellular pathogen sensors that recognize PRRSV have not been elucidated yet (14–20). The purpose of this work is to identify whether the intracellular pathogen sensor DHX36 is involved in PRRSV-induced inflammatory response. In this study, we found that the expression levels of DHX36 in cytoplasm increase after PRRSV infection. Knockdown and overexpression analyzes showed that DHX36 is involved in both PRRSV and N protein-induced NF-κB signal activation. Furthermore, co-immunoprecipitation confirmed that N protein binds to DHX36, both in transfected HEK293T cells and in PRRSV-infected MARC-145 cells. Thus, our studies have illuminated a cellular mechanism responsible for PRRSV-associated inflammatory responses that may contribute to a deeper understanding of the infection and pathogenesis of PRRSV.

## Materials and Methods

### Virus, Cells, and Reagents

Porcine reproductive and respiratory syndrome virus WUH3 strain (GenBank accession number: HM853673.2), isolated from the brain of pigs suffering from “high fever” syndrome in China at the end of 2006 and identified as a highly pathogenic North American type PRRSV (type 2), was amplified and titrated as described previously (21). UV-inactivated PRRSV was irradiated 30 cm under short-wave (254 nm) ultraviolet light (40 W) for 1 h. Loss of infectivity was confirmed by the inability of the UV light-exposed viruses to produce cytopathic effect on monolayers of MARC-145 cells. MARC-145 cells were grown and propagated in Dulbecco’s Modified Eagle medium (Invitrogen, Carlsbad, CA, USA) supplemented with 10% heat-inactivated fetal bovine serum, at 37°C in a humidified 5% CO_2_ incubator. HEK293T cells were cultured and maintained in RPMI-1640 (Invitrogen) supplemented with 10% heat-inactivated fetal bovine serum. Porcine primary pulmonary macrophages (PAMs) used in this study have been described previously (22).

Mouse monoclonal anti-hemagglutinin (anti-HA), anti-FLAG, and anti-β-actin antibodies were purchased from ABclonal Biotechnology. Anti-FLAG polyclonal antibody (Macgene, China), anti-DHX36 polyclonal antibody (Proteintech, China), anti-TRIF polyclonal antibody (Proteintech, China), anti-MyD88 polyclonal antibody (Proteintech, China), anti-HSP90 polyclonal antibody (Proteintech, China), anti-LaminA + C polyclonal antibody (Proteintech, China), anti-IκBα, anti-NF-κB P65, and anti-phosphor-p-P65 polyclonal antibodies (Cell Signaling) were purchased and used according to the manufacturers’ recommendations. Horseradish peroxidase-conjugated anti-mouse or anti-rabbit IgG antibodies were purchased from Beyotime Institute of Biotechnology (Jiangsu, China). PRRSV N protein monoclonal antibody was produced from hybridoma cells derived from Sp2/0 myeloma cells and spleen cells of BALB/c mice immunized with recombinant N protein from PRRSV strain WUH3.

### Plasmids

The FLAG or HA epitope tag was amplified by PCR and cloned into the pCAGGS-MCS vector to generate the pCAGGS-FLAG or pCAGGS-HA plasmid, encoding an N-terminal FLAG or HA tag. Expression plasmids for FLAG-tagged DHX36 were constructed by PCR amplification of the cDNA from PAMs. Plasmids encoding truncated DHX36 were constructed by PCR amplification using the specific primers listed in Table S1 in Supplementary Material. The HA-tagged expression plasmid encoding the PRRSV N protein (pCAGGS-HA-N) used in this study, and its expression was confirmed with HA-tag immunoblotting (23). Reporter plasmids NF-κB reporter plasmid and pRL-TK were described elsewhere (21). All constructs were confirmed by DNA sequencing.

### RNA Extraction and Real-time RT-PCR

MARC-145 cells grown in 24-well plates were infected with PRRSV or mock-infected at an MOI of 1. Total RNA was isolated at the indicated time points using TRIzol reagent (Invitrogen). Real-time RT-PCR was performed using SYBR Green Real Time PCR Master Mix (Toyobo Biologics, Osaka, Japan) in a LightCycler 480 (Roche Molecular Biochemicals). Individual transcripts in each sample were assayed three times. The PCR conditions were as follows: initial denaturation for 10 min at 95°C, followed by 40 cycles of 15 s at 95°C, 15 s at 58°C, and 40 s at 72°C. The fold change in gene expression relative to normal was calculated using the delta delta cycles to threshold (ΔΔCT) method (24). Primers (Table S2 in Supplementary Material) were designed using the Primer Express software (version 3.0; Applied Biosystems, Carlsbad, CA, USA).

### Transfection and Luciferase Reporter Assay

Transient transfection was carried out using Lipofectamine 2000 (Invitrogen). MARC-145 cells were seeded on 24-well plates at a density of 2–4 × 10^5^ cells/well and cultured until the cells reached approximately 70–80% confluence. Cells were then transfected with the indicated plasmids or small-interfering RNA (siRNA) in triplicate. For each transfection, 0.2 µg of the NF-κB reporter plasmid (purchased from Stratagene) along with 0.05 µg pRL-TK and 80 nM siRNA were used. Firefly and *Renilla* luciferase activities were determined using the dual-luciferase reporter assay system (Promega) according to the manufacturer’s instructions. siRNA sequences used are as follows: si-DHX36, sense 5′-GGAGCCGGAUUUGUAAGCAGUAGAA-3′, antisense 5′-UUCUACUGCUUACAAAUCCGGCUCC-3′; si-MyD88, sense 5′-CUGGUCCAUUGCUAGUGAATT-3′, antisense 5′-UUCACUAGCAAUGGACCAGTT-3′; si-TRIF, sense 5′-GACACCACCUCUCCAAAUATT-3′, antisense 5′-UAUUUGGAGAGGUGGUGUCTT-3′; negative control (NC) siRNA, sense 5′-UUCUCCGAACGUGUCACGUTT-3′, antisense 5′-ACGUGACACGUUCGGAGAATT-3′.

### Western Blotting Analysis

Cytoplasm and nuclear protein extracts from PRRSV-infected PAMs or HEK293T cells after transfection with N protein expression plasmid were prepared with the cytoplasmic and nuclear protein extraction kit (Aidlab Biotechnologies Co., Ltd.) according to the manufacturer’s protocols. Cells cultured in 60-mm dishes were prepared by adding 120 µL 2× lysis buffer A (65 mM Tris-HCl, pH 6.8, 4% sodium dodecyl sulfate (SDS), 3% dl-dithiothreitol, and 40% glycerol). The cell extracts were boiled for 10 min and then resolved with 8–12% SDS-PAGE. The separated proteins were electroblotted (Bio-Rad Transblot Cell System, USA) onto a polyvinylidenedifluoride (PVDF) membrane (Millipore, Billerica, MA, USA) and run for 3–5 h at 40 V on ice. The Western blot was probed with specific antibodies, and the expression of β-actin was detected with an anti-β-actin mouse monoclonal antibody to demonstrate equal protein sample loading.

### Co-Immunoprecipitation and Immunoblotting Analyses

To investigate the interactions between proteins, HEK293T cells or MARC-145 cells were lysed in immunoprecipitation lysis buffer (RIPA). After the lysate was clarified by centrifugation at 12,000 × *g* for 10 min, the lysate proteins were incubated overnight at 4°C with the indicated antibodies. Protein A + G agarose beads (30 µl; Beyotime) were then added to each immunoprecipitation reaction for another 6 h. The agarose beads were then washed three times and the captured proteins resolved on 8–12% SDS-PAGE, transferred to PVDF membranes, and analyzed by immunoblotting.

### Indirect Immunofluorescence Assay

MARC-145 cells seeded on microscope coverslips and placed in 24-well dishes were infected with PRRSV (MOI = 0.5). At 24 hpi, the cells were fixed with 4% paraformaldehyde for 10 min and then permeated with 0.1% Triton X-100 for 10 min at room temperature. After three washes with PBS, the cells were sealed with PBS containing 5% bovine serum albumin for 1 h and then incubated separately with rabbit polyclonal antibody directed against DHX36 (1:200) and mouse monoclonal antibody directed against PRRSV N protein (1:200) for 1 h at 37°C. The cells were then treated with fluorescein-isothiocyanate-labeled goat anti-mouse or Cy3-labeled goat anti-rabbit antibodies (Invitrogen) for 1 h, followed by 4′,6-diamidino-2-phenylindole for 10 min at room temperature. After the samples were washed with PBS, fluorescent images were acquired with a confocal laser scanning microscope (Olympus Fluoview ver. 3.1, Japan).

### Statistical Analysis

The statistical analyses (Student’s *t* test) and generation of graphs were performed with the GraphPad Prism^®^ 6 software. Data are presented as mean ± standard deviation. *P < 0.05, **P < 0.01.

## Results

### PRRSV Infection Steadily Increases the Expression of DHX36

**Figure 1:**
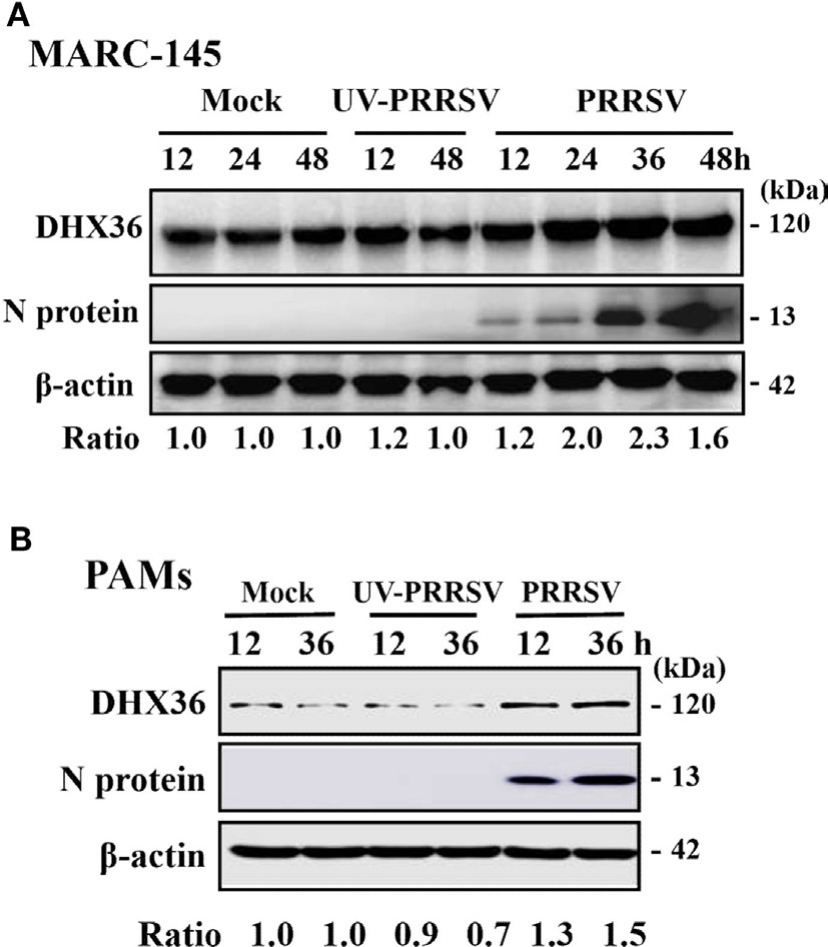
DExD/H-box helicase 36 (DHX36) expression is modulated by porcine reproductive and respiratory syndrome virus (PRRSV) but not UV-inactivated PRRSV in MARC-145 cells and pulmonary macrophages (PAMs). MARC-145 cells **(A)** and PAMs **(B)** were mock-infected, infected with PRRSV, or UV-inactivated PRRSV at an MOI of 1. Cell lysates were collected at the indicated time points and the expression of endogenous DHX36 protein was analyzed by Western blot with DHX36-specific antibody. Relative levels of DHX36 in comparison with mock-infected cells are indicated as fold change below the images. Western blot with a specific monoclonal antibody against PRRSV N protein demonstrates PRRSV infection. All blots were also incubated with anti-actin antibody to verify equal protein loading.

To better understand the role of DHX36 in PRRSV infection, we first examined the expression levels of endogenous DHX36 after PRRSV infection. MARC-145 cells were mock-infected or infected with PRRSV, and cells were collected at 12, 24, 36, and 48 h post-infection (hpi) for Western blotting analyses. As shown in Figure 1A, elevated endogenous DHX36 protein was first observed at 24 hpi in PRRSV-infected MARC-145 cells and achieved stronger intensity at 36 hpi, while stimulation with UV-inactivated PRRSV failed to alter DHX36 protein expression, indicating that the up-regulation of DHX36 depends on viral replication.

To substantiate that the expression of DHX36 is induced by PRRSV, we then tested the expression of DHX36 in PAMs. To this end, PAMs were mock-infected or infected with PRRSV or UV-inactivated PRRSV and collected at 12 and 36 hpi before subjected to Western blot analyses. As shown in Figure 1B, the expression of DHX36 was increased in PRRSV-infected PAMs. UV-inactivated PRRSV also failed to alter DHX36 protein expression. Collectively, above data support the basal presence of DHX36 in unstimulated cells and demonstrated that DHX36 is modulated by PRRSV infection.

### DHX36 Resided Mainly in the Cytoplasm after PRRSV Infection

**Figure 2:**
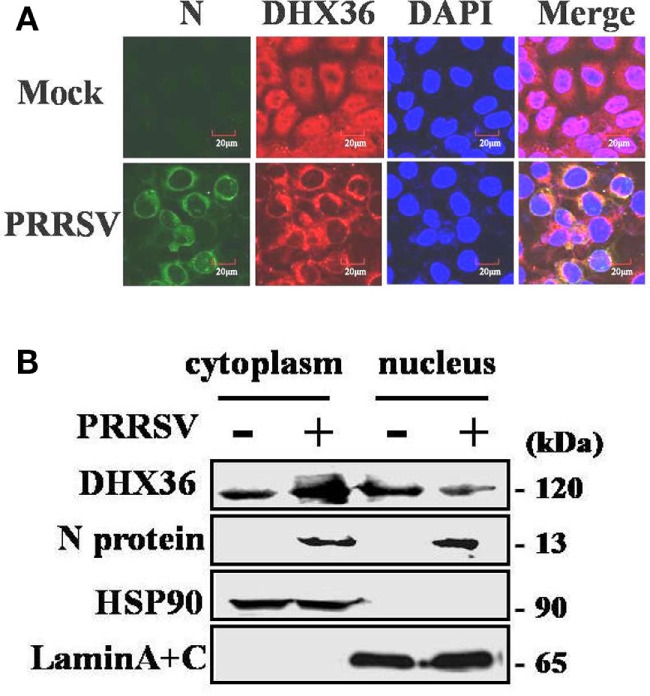
Effect of porcine reproductive and respiratory syndrome virus (PRRSV) infection on the subcellular distribution of DExD/H-box helicase 36 (DHX36). **(A)** MARC-145 cells were mock-infected or infected with PRRSV (MOI = 0.5). At 24 hpi, cells were fixed for immunofluorescence analysis of DHX36 (red), N protein (green), and nucleus marker diamidino-2-phenylindole (blue) localization and merged by confocal microscopy. **(B)** Nuclear and cytoplasmic fractionation of PAMs infected with PRRSV for 24 h. Each nuclear and cytosolic fraction was prepared from PAMs and subjected to Western blot analysis with an antibody specific for DHX36, LaminA + C as a nuclear protein marker, HSP90 as a cytosolic protein marker, or the PRRSV N protein.

The subcellular localization of DExD/H-box helicase protein in host cell is consistent with its function. DHX36 is a nucleocytoplasmic shuttling protein involved in a wide range of cellular functions both in the cytoplasm and nucleus (3). To explore the subcellular localization of DHX36 after PRRSV infection, MARC-145 cells were inoculated with PRRSV for 24 h, and indirect immunofluorescence assay was performed with a polyclonal antibody against DHX36. The PRRSV-infected cells were monitored by monoclonal antibody against viral N protein. As shown in Figure 2A, endogenous DHX36 protein was distributed diffusely in both cytoplasm and cell nuclei in mock-infected cells; in contrast, even though some of the DHX36 still resided in the nucleus, PRRSV infection resulted in the accumulation of DHX36 in the cytoplasm.

To further reinforce this observation, the nuclear and cytoplasmic fractions were separated from PAMs at 24 h after PRRSV infection, followed by Western blot analyses. As expected, HSP90 and Lamin A + C, the cytoplasmic and nuclear protein markers remained unchanged in their fractions, respectively, eliminating the possibility of fractions cross-contamination and unequal sample loading (Figure 2B). In accord with the results in MARC-145 cells, the increased DHX36 mainly located in cytoplasmic fraction in PRRSV-infected PAMs (Figure 2B). These results suggest a possibility that PRRSV infection might provide potential ligand, which redistributes DHX36 into the cytoplasm.

### The DHX36–MyD88 Axis Is Involved in PRRSV-Induced NF-κB Activation

**Figure 3:**
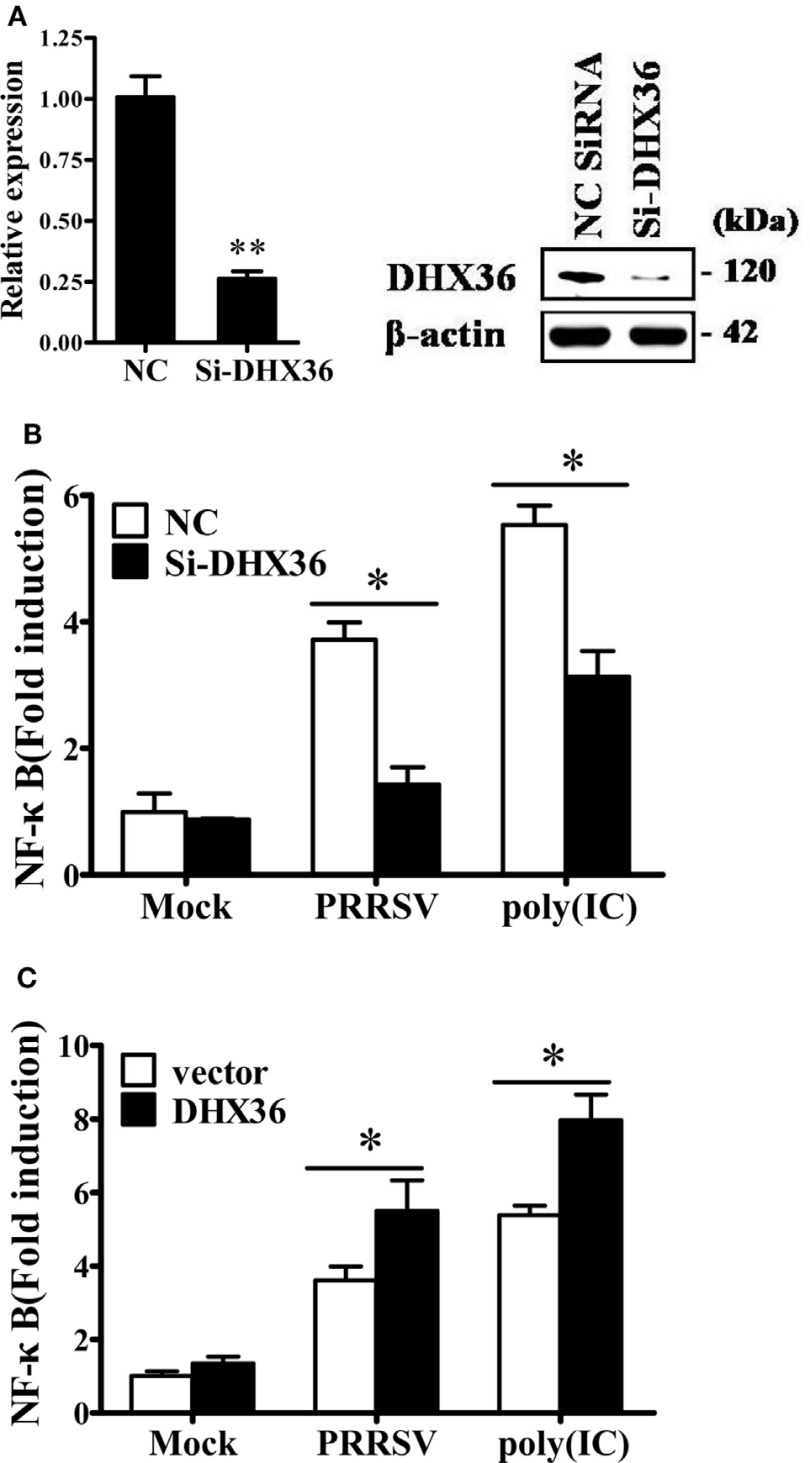
DExD/H-box helicase 36 (DHX36) is involved in porcine reproductive and respiratory syndrome virus (PRRSV)-induced NF-κB activation in MARC-145 cells. **(A)** MARC-145 cells were transfected with small interfering RNA (siRNA)-targeting DHX36 or control siRNA. Thirty-six hours later, real-time RT-PCR and Western blot were performed to analyze the mRNA and protein expression of DHX36. **(B)** MARC-145 cells were co-transfected with the NF-κB reporter plasmid, pRL-TK plasmid, along with the indicated siRNA. At 24 h after transfection, cells were stimulated by poly I:C (1 µg/ml) or infected with PRRSV (MOI = 1) for 24 h. Cells were then collected and lysed for dual-luciferase assay. **(C)** MARC-145 cells were transiently transfected with either DHX36 or control plasmid along with NF-κB luciferase reporter plasmid. At 36 h post-transfection, cells were stimulated with mock, PRRSV (MOI = 1) or poly I:C (1 µg/ml, transfected with Lipofectamine) followed by luciferase assay.

In cytoplasm, DHX36 plays important roles in sensing poly I:C and viral infection (3, 25). Considering that pigs infected with PRRSV may develop severe interstitial pneumonia, we focused on the relationship between DHX36- and PRRSV-induced pro-inflammatory NF-κB signaling activation. First, we utilized DHX36-specific siRNA to silence DHX36. The knockdown efficiency of the synthesized siRNA-targeting DHX36 (si-DHX36) was evaluated by real-time RT-PCR and Western blotting analyses. Compared with the cells treated with NC siRNA, cells transfected with si-DHX36 significantly decreased DHX36 expression at protein and mRNA levels (Figure 3A). Next, we assessed the impact of DHX36 silencing in response to PRRSV stimulation. Poly I:C was used as a positive control. As expected, when DHX36 was knocked down in MARC-145 cells, the poly I:C-induced activation of NF-κB-promoter-dependent luciferase activity was less pronounced (Figure 3B). Similarly, compared to DHX36-knockdown MARC-145 cells, cells transfected with NC siRNA induced high levels of NF-κB activation following stimulation with PRRSV, highlighting the importance of DHX36 for NF-κB signal activation in response to PRRSV infection (Figure 3B).

To further validate the function of DHX36 in PRRSV-induced activation of NF-κB, we again stimulated NF-κB promoter activity using PRRSV after DHX36 overexpression. Synthetic RNA duplex poly I:C was also included for comparison. Under the same conditions, overexpression of DHX36 significantly augmented the poly I:C- and PRRSV-induced activation of the NF-κB promoter as compared with empty vector-transfected cells (Figure 3C).

**Figure 4:**
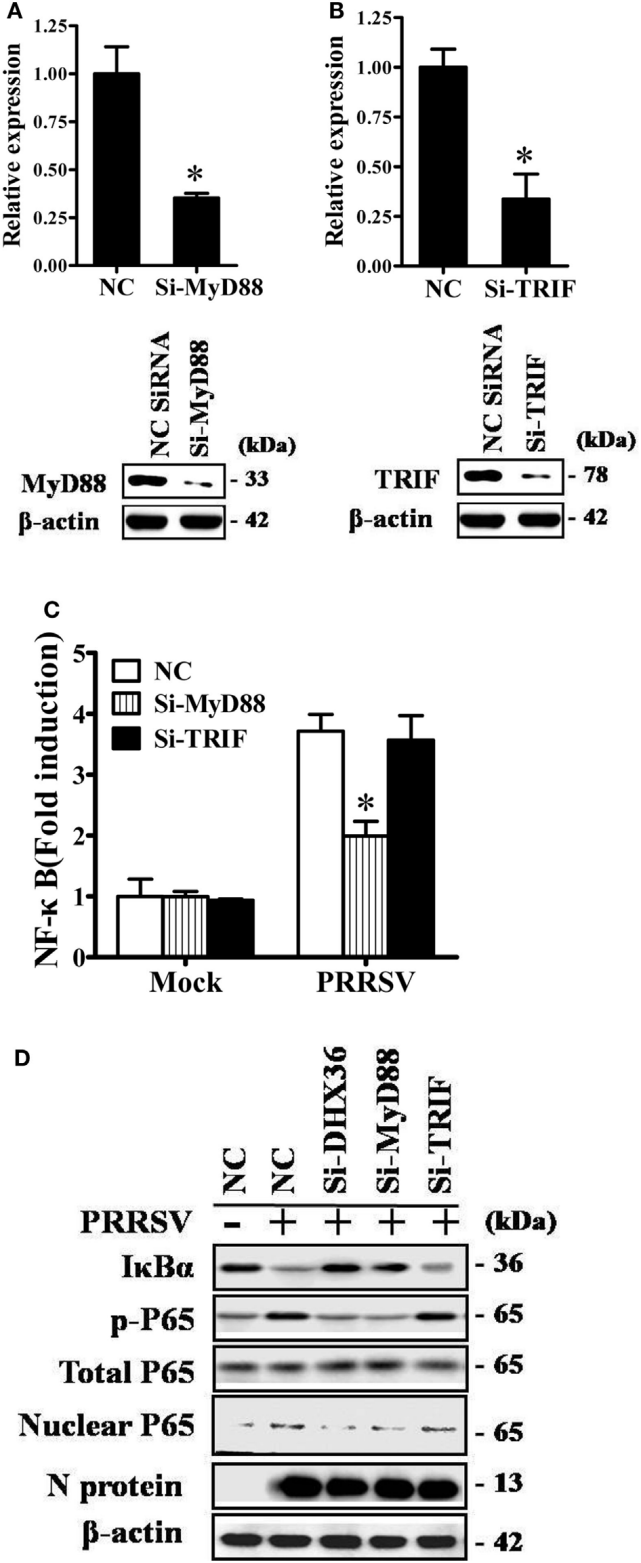
DExD/H-box helicase 36 (DHX36) utilizes myeloid differentiation primary response gene 88 (MyD88) to activate NF-κB signaling in porcine reproductive and respiratory syndrome virus (PRRSV)-infected MARC-145 cells. **(A,B)** Silencing efficiency of small-interfering RNA (siRNA) targeting MyD88 **(A)** or TIR-domain-containing adapter-inducing IFN-β (TRIF) **(B)**. MARC-145 cells were transfected with the indicated siRNA. Thirty-six hours later, real-time RT-PCR and Western blot were performed to analyze the mRNA and protein expression of MyD88 or TRIF. **(C)** MARC-145 cells were transfected with the indicated siRNA along with the NF-κB reporter plasmid and pRL-TK plasmid. At 24 h after transfection, cells were infected with PRRSV (MOI = 1) for 24 h before being lysed for dual-luciferase assay. **(D)** MARC-145 cells were transfected with si-DHX36, si-MyD88, or control siRNA. At 36 h after transfection, cells were infected with PRRSV (MOI = 1). Cytoplasmic and nuclear extracts were prepared at 36 h post-infection. The levels of IκBα, phosphorylated P65, total P65, and β-actin were evaluated by Western blotting at 36 hpi, and the blot was probed with a specific monoclonal antibody directed against PRRSV N protein to confirm PRRSV infection.

Two adaptor molecules, TRIF and MyD88, have been reported to be utilized by DHX36 to mediate downstream signaling (2, 3). To further identify which adaptor is utilized by PRRSV to induce NF-κB activation, we again used siRNA to knock down endogenous MyD88 or TRIF and confirmed the knockdown efficiency by real-time RT-PCR and Western blotting analyses (Figures 4A,B). As shown in Figure 4C, compared with NC siRNA, knockdown of MyD88 significantly reduced PRRSV-induced NF-κB activation, but no appreciable change was detected after knockdown of TRIF. We further validated this observation by Western blotting analysis of IκBα expression level and P65 phosphorylation. To this end, MARC-145 cells were transfected with DHX36-, MyD88-, and TRIF-specific siRNA or control scrambled siRNA, after which the cells were infected with PRRSV, and cell extracts were prepared and separated by SDS-PAGE, followed by immunoblotting. Consistent with the results of the dual-luciferase assay, Western blotting showed that IκBα protein degradation and P65 phosphorylation were strongly impaired in both DHX36- and MyD88-knockdown cells, whereas these parameters barely changed in TRIF-knockdown cells (Figure 4D). These results indicate that MyD88 is the key downstream adaptor of DHX36 responsible for PRRSV-induced NF-κB activation.

### DHX36 and MyD88 Silencing Impair PRRSV-Induced Expression of NF-κB-Dependent Pro-inflammatory Cytokines

**Figure 5:**
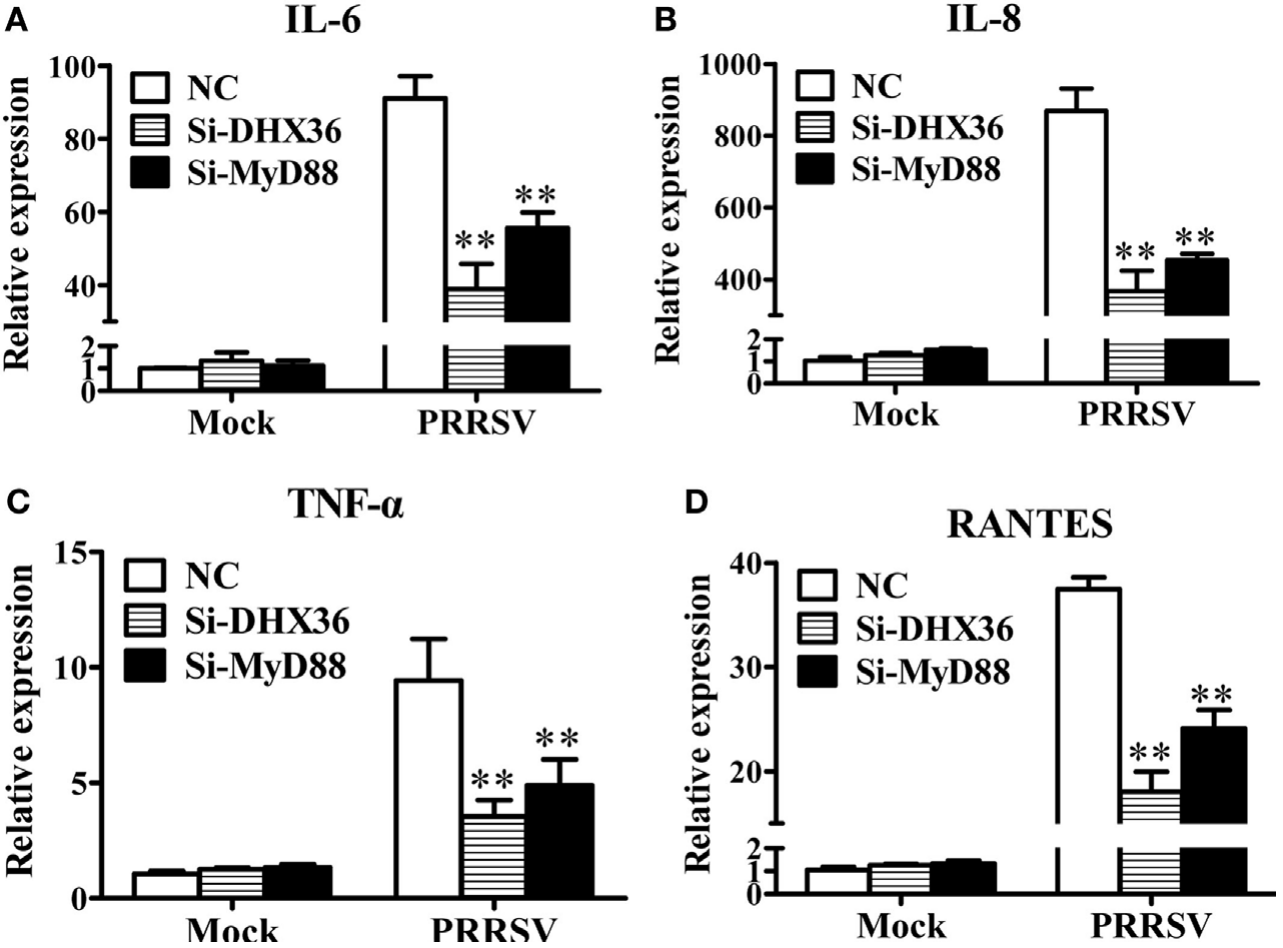
The DExD/H-box helicase 36 (DHX36)–MyD88 axis is involved in porcine reproductive and respiratory syndrome virus (PRRSV)-induced production of pro-inflammatory cytokines. **(A–D)** MARC-145 cells were transfected with si-DHX36, si-MyD88 or negative control small interfering RNA for 24 h prior to PRRSV (MOI = 1) infection for 36 h. The mRNA expression of IL-6 **(A)**, IL-8 **(B)**, TNF-α **(C)**, and RANTES **(D)** was evaluated by real-time RT-PCR.

NF-κB is the key transcription factor for pro-inflammatory cytokine production following PRRSV infection (26–28). DHX36 is involved in PRRSV-induced NF-κB activation, thus theoretically, it should be associated with PRRSV-induced cytokine production. To test this, MARC-145 cells were transfected with DHX36-specific siRNA to knock down endogenous DHX36 expression, and then infected with PRRSV. At 36 hpi, cells were collected for real-time RT-PCR to analyze the expression of various cytokines. As shown in Figures 5A–D, silencing of endogenous DHX36 led to drastic reduction of IL-6 (Figure 5A), IL-8 (Figure 5B), TNF-α (Figure 5C), and RANTES (Figure 5D) mRNA expression in PRRSV-treated MARC-145 cells. Accordingly, knockdown of MyD88 also resulted in >40% loss in cytokine production (Figures 5A–D), indicating that DHX36 is critical for PRRSV-induced cytokine transcription and that DHX36-driven pro-inflammatory cytokine expression is dependent on MyD88.

### PRRSV N Protein-Induced NF-κB Activation Involves DHX36

**Figure 6:**
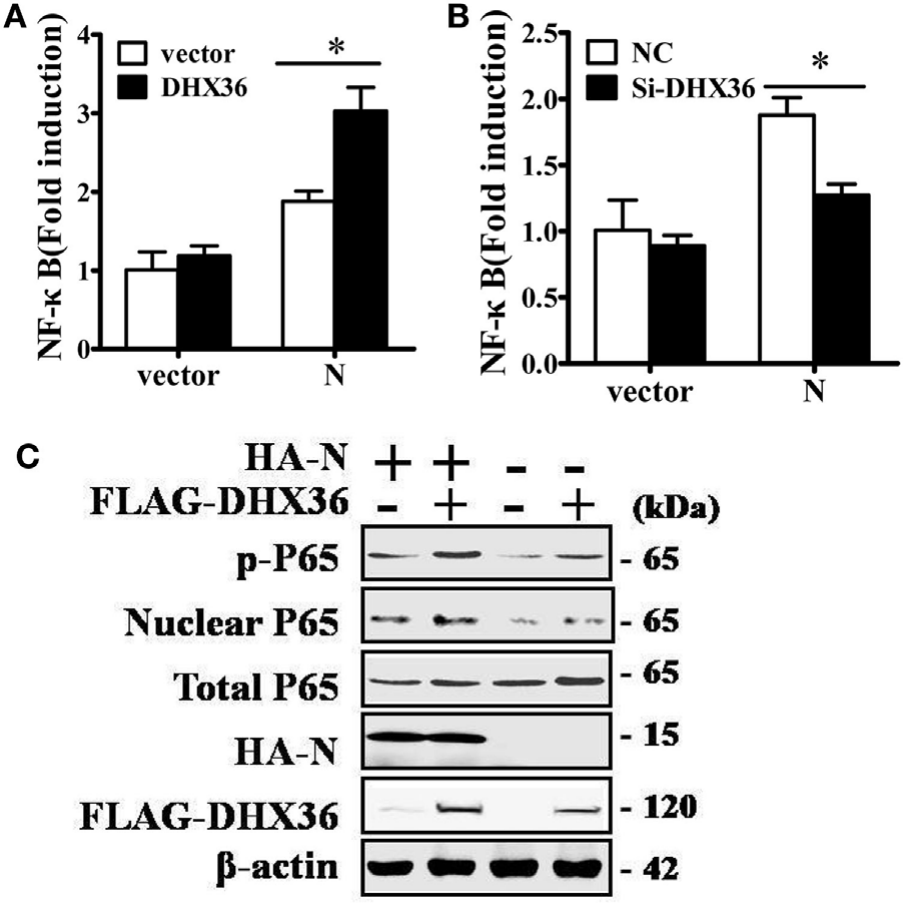
DExD/H-box helicase 36 (DHX36) is involved in N protein-induced NF-κB activation. **(A)** MARC-145 cells were transfected with the N protein and/or DHX36 along with NF-κB reporter plasmid and the pRL-TK plasmid. Cells were collected for dual-luciferase assay at 36 h post-transfection. **(B)** MARC-145 cells were transfected with either DHX36 small-interfering RNA (siRNA) or negative control siRNA. At 24 h post-transfection, cells were transfected with NF-κB reporter plasmid and pRL-TK plasmid along with N protein. After a further 36 h, cells were collected and lysed for dual-luciferase assay. **(C)** HEK293T cells were co-transfected with expression vectors encoding FLAG-DHX36 and/or HA-N. Cytoplasmic and nuclear extracts were prepared at 28 h post-transfection and subjected to Western blot analysis with antibodies specific for P65 or p-P65.

Among PRRSV-encoded proteins, the N protein has been identified as the key NF-κB activator (23). To further investigate whether DHX36 is involved in N protein-induced NF-κB signal activation, pCAGGS-HA-N, pCAGGS-FLAG-DHX36, pRL-TK, and an NF-κB reporter plasmid were transiently co-transfected into MARC-145 cells. After 36 h transfection, a dual-luciferase assay was performed to detect NF-κB reporter activation. As shown in Figure 6A, N protein overexpression could initiate the transcription of an NF-κB promoter-driven luciferase construct and overexpression of DHX36 further augmented N protein’s ability to activate NF-κB. We also evaluated the involvement of DHX36 in N protein-induced NF-κB activation by knockdown of endogenous DHX36. As shown in Figure 6B, we observed that loss of DHX36 led to a significant decrease in the ability of N protein to activate luciferase under control of the NF-κB promoter.

To further confirm NF-κB activation, we examined the phosphorylation and nuclear translocation of NF-κB subunit P65 in cells co-expression of N protein and DHX36. As shown in Figure 6C, the amount of phosphorylated P65 and nuclear P65 protein increased, while total P65 was unaltered in cells co-expressing N protein and DHX36, compared to cells expressing only N protein. These results indicate that forced expression of N protein in MARC-145 cells significantly activates NF-κB signaling and this effect involves DHX36.

### DHX36 Interacts with N Protein *via* N-Terminal Domain

**Figure 7:**
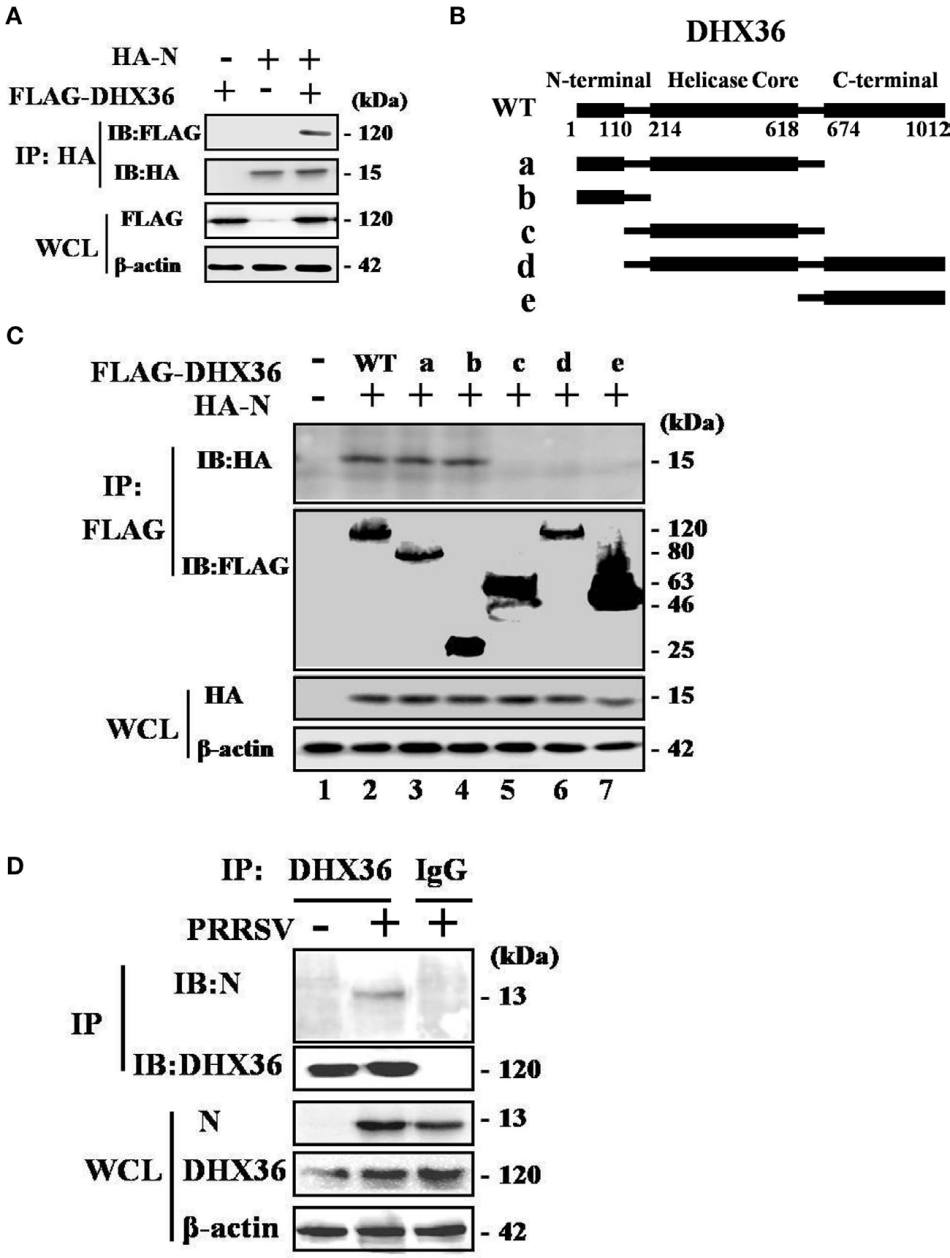
Porcine reproductive and respiratory syndrome virus (PRRSV) N protein interacts with DExD/H-box helicase 36 (DHX36). **(A)** HEK293T cells were co-transfected with expression vectors encoding FLAG-DHX36 and/or HA-N. Cell lysates were co-immunoprecipitated with an anti-hemagglutinin antibody and immunoblotted with an anti-FLAG antibody to assess the interaction between DHX36 and N protein. **(B)** Schematic representation of DHX36 mutants. Structurally, DHX36 consists of a highly conserved catalytic core (214–618 aa) flanked by ancillary N-terminal region (1–110 aa) and C-terminal region (674–1012 aa), which have been shown to provide substantial substrate specificity through interaction with RNA or proteins. **(C)** CO-IP and immunoblot analysis of HEK293T cells transfected with deletion mutants of FLAG-DHX36 along with vector for HA-N. **(D)** Anti-DHX36 immunoprecipitation and immunoblot analysis of extracts from MARC-145 cells infected with PRRSV for 24 h. Cell lysates were also immunoprecipitated with rabbit normal IgG as control.

To test whether PRRSV N protein might function as a ligand of the intracellular sensor DHX36 to initiate NF-κB signaling activation in mammalian cells, we transfected HEK293T cells with FLAG-tagged DHX36 and HA-tagged N protein then examined their interaction. As shown in Figure 7A, DHX36 was detected in anti-HA co-immunoprecipitates from N protein co-transfectants but not from cells co-transfected with the control plasmid, indicating that FLAG-tagged DHX36 forms a complex with HA-tagged N protein in co-immunoprecipitation assays.

Full-length porcine DHX36 cDNA comprises 1012 amino acid residues, containing a helicase core domain and is flanked on either side by N- and C-terminal extensions (Figure 7B). To examine which domain of DHX36 might be involved in N protein binding, five mutants with deletion of different domains of DHX36 were constructed by mutagenesis (Figure 7B). HEK293T cells were co-transfected with various combinations of FLAG-tagged full length or deleted versions of DHX36 and HA-tagged N protein. As shown in Figure 7C, the CO-IP experiments indicated that mutants with deletion of the N-terminal domain of DHX36 were incapable of interacting with PRRSV N protein.

Finally, we examined whether N protein interacted with endogenous DHX36 after PRRSV infection. To address this, MARC-145 cells were mock infected or infected with PRRSV (MOI = 0.5) for 24 h before cells were harvested. The endogenous DHX36 was then precipitated, and immunoblotting was performed with an anti-N monoclonal antibody. As shown in Figure 7D, CO-IP analysis indicated that N protein associated with DHX36 in PRRSV-infected MARC-145 cells. Besides, cytoplasmic fractions contained both DHX36 and N protein in PRRSV-infected cells, which provided evidence of their colocalization (Figures 2A,B). Collectively, the above results indicate that DHX36 recognizes N protein and contributes to both PRRSV- and N protein-induced NF-κB pathway activation.

## Discussion

**Figure 8:**
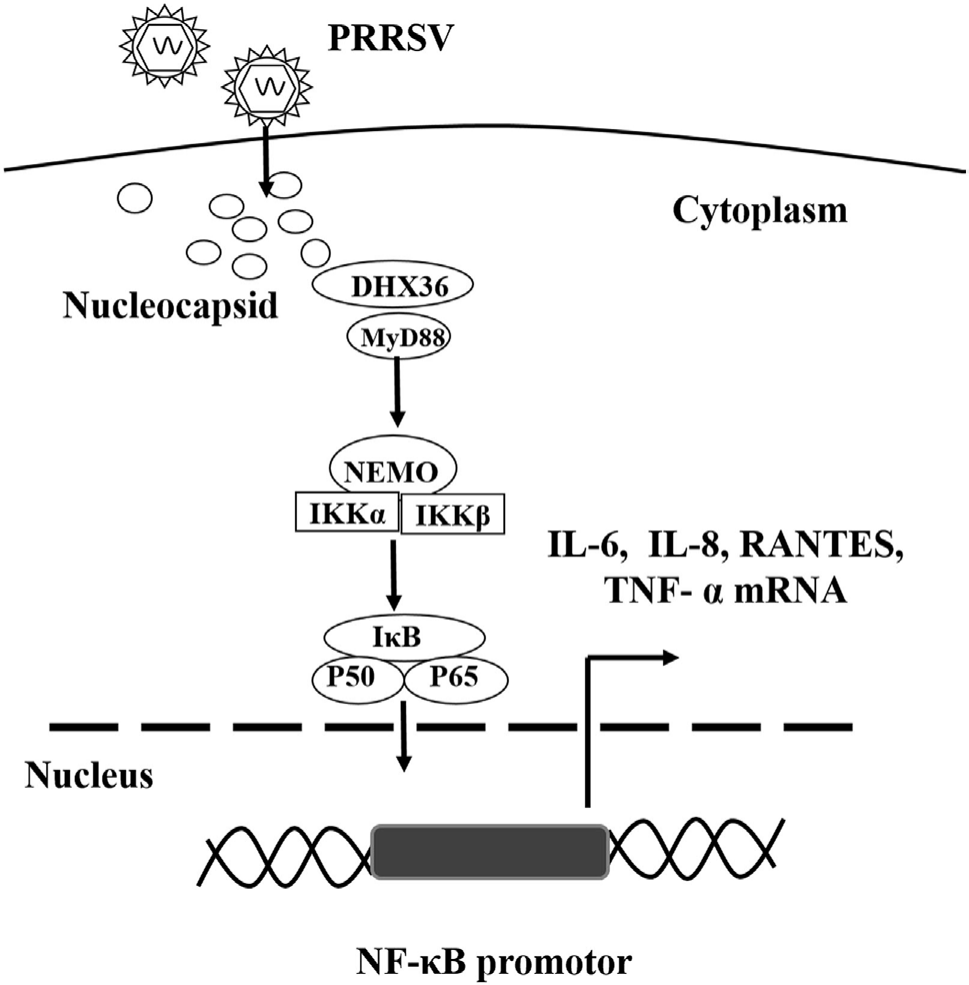
Proposed model illustrating the DExD/H-box helicase 36 (DHX36)–MyD88 axis involvement in PRRSV N protein-induced NF-κB signaling. PRRSV infection induces the up-regulation and relocalization of DHX36 from the nucleolus to the cytoplasm. DHX36 in the cytoplasm senses the N protein of PRRSV and then recruits MyD88 to facilitate P65 phosphorylation, which in turn initiates NF-κB-dependent IL-6, IL-8, TNF-α and RANTES transcription.

Modulation of the inflammatory response in the respiratory tract contributes significantly to PRRSV pathogenesis as well as lung disease severity, but the mechanism is poorly understood (6, 15, 29, 30). In this study, we demonstrate that PRRSV infection significantly increases NF-κB-driven inflammatory cytokine production by activating the DHX36–MyD88-P65 signaling cascade. Knockdown of DHX36 inhibits both PRRSV- and N protein-induced activations of the NF-κB signaling. Our findings suggest a new mechanism through which PRRSV employs a viral nucleocapsid protein to initiate DHX36-dependent innate responses to promote activation of NF-κB pathway (Figure 8).

Nucleocapsid protein has been identified as the key NF-κB activator among PRRSV-encoded proteins in previous studies (23, 31); however, it remains unclear, which PRRs recognize this protein and contributes to NF-κB pathway activation. In fact, host cells possess a number of PRRs to detect invading virus (28). Viral proteins in some cases can serve as PAMPs to be recognized by certain PRRs such as TLR2 and TLR4, to trigger the innate immune response (32, 33). In this study, we demonstrate that the N protein of PRRSV binds to DHX36 in both transfected HEK293T cells and PRRSV-infected MARC-145 cells. The interactome map of the PRRSV N protein has been examined using a proteomics approach, and several DExD/H-box helicase superfamily proteins, including DDX3X, DDX5, DDX17, DHX9, DHX30, and DHX36, were identified (34, 35). Consistent with this observation, our work not only validated the interaction between N protein and DHX36 but further showed that the N-terminal domain of DHX36 was responsible for mediating binding of PRRSV N protein. In addition, inhibition of DHX36 expression by specific siRNA impaired N protein-mediated NF-κB activation. Our study therefore provides evidence that a virus-derived protein can bind to the cytoplasmic PRR DHX36 and can subsequently stimulate NF-κB activation.

During the last decade, several reports have illustrated differential functions of DExD/H-box helicase superfamily proteins in PRRSV infection. For example, overexpression of DDX3X was found to significantly inhibit PRRSV replication in MARC-145 cells (36). In addition, it has been shown that DDX5 plays a positive role in the replication of PRRSV *via* its interaction with viral nsp9 in PRRSV-infected MARC-145 and PAM cells (37). More recently, DHX9 was reported to be recruited by the N protein during PRRSV infection to aid the synthesis of viral genomic RNA (38). Although the roles of DExD/H-box family proteins in viral replication are well documented, their importance in detecting invading virus is just becoming evident. In this regard, DDX19a was discovered to be an RNA sensor that triggers NLRP3 inflammasome activation and IL-1β production by detecting PRRSV genomic RNA (4). Thus, both DDX19a-NLRP3-related viral RNA sensing and DHX36-mediated viral N protein recognition might contribute collaboratively to uncontrolled progression of virus-induced inflammation, which culminates in exacerbated pathogenesis and development of pneumonia.

DExD/H-box helicase 36 was originally identified as crucial for the maintenance of genomic integrity, prevention of abortive transcription, and translation initiation (5, 39, 40). As an evolutionarily conserved DEAH-box ATP-dependent helicase, DHX36 is highly specific for DNA and RNA G4s with N-terminal extension motifs required for quadruplex recognition (5). This N-terminal domain was also found to be both essential and sufficient for its localization in stress granules, the membraneless RNA- and RNA-binding protein-containing complexes that are transiently assembled in stressful conditions to promote cell survival (41). Recently, biochemical data have shown that DHX36 and double-stranded RNA-activated protein kinase (PKR) form a complex in a dsRNA-dependent manner and facilitate dsRNA binding, phosphorylation, and activation of PKR, processes which are essential for the formation of stress granules (25). It is noteworthy that stress granule formation is also triggered by PRRSV infection, which in turn facilitates pro-inflammatory cytokine production (unpublished data). These observations imply that there is some unidentified relationship between PRRSV-triggered stress granule formation and the activation of DHX36 signaling. In addition, Western blots were performed to detect the expression of P65 and p-P65 by co-expression of N protein and the wild-type DHX36 or individual DHX36 mutant. We found that, except the wild-type DHX36, all DHX36 mutants (mutants a, b, c, d, and e) cannot up-regulate the phosphorylation level of P65 when co-expressed with N protein (data not shown). This is possibly because DHX36 mutants a, b, and c lack the C-terminal MyD88-binding domain responsible for downstream signaling activation, while mutants d and e lose the N-terminal domain responsible for binding with PRRSV N protein.

Viral-derived proteins are frequently documented as negative regulators of innate immunity (42–49). Consistent with this, it has been shown that the PRRSV N protein can suppress IFN production by inhibiting IRF3 phosphorylation and nuclear translocation (43). Among the viral-derived proteins that are involved in PRRSV-induced pathogenesis, identification of the major contributor to severely impaired IFN responses and the massive inflammation in the respiratory tract associated with PRRSV infection, along with dissection of the underlying molecular mechanism, is of critical importance. Although recent studies have revealed that several proteins encoded by the PRRSV genome independently antagonize IFN signaling, only N protein has been widely accepted to possess the ability of activating NF-κB signaling (23). Thus, the current study sought to identify the mechanisms by which PRRSV N protein activates NF-κB-driven inflammatory responses and identified that DHX36 is involved in N protein-mediated NF-κB activation. In fact, the N protein of PRRSV is a multifunctional protein in addition to its role in packaging viral genomic RNA (9, 50). Several amino acid motifs on the N protein have been identified not only to be involved in virus replication but are also associated with cellular proteins and signaling pathways (51, 52), such as translation initiation and RNA post-transcriptional modification (53–55). These studies, together with our work, demonstrate the importance of the N protein in the life cycle of *Arteriviruses*.

In summary, our present study confirms that the expression of DHX36 is markedly induced in PRRSV-infected MARC-145 cells. Knockdown of DHX36 significantly suppresses the NF-κB-driven pro-inflammatory cytokine production by PRRSV infection. Moreover, DHX36 interacts with the N protein of PRRSV and is involved in N protein-induced activation of NF-κB signaling. This work provides a new understanding of the ability of type 2 PRRSV to induce NF-κB activation, but whether DHX36 is also associated with the pathogenesis of PRRSV EU strain (type 1) deserves further investigation.

## Author Contributions

SX, HC, and HJ designed the experiments. HJ, YZ, ZD, and WK performed the experiments. HJ and DW analyzed the data. HJ, SX, and LF wrote the paper. All the authors read and approved the manuscript.

## Conflict of Interest Statement

The authors declare that the research was conducted in the absence of any commercial or financial relationships that could be construed as a potential conflict of interest.

## References

[1] Chan YK Gack MU. Viral evasion of intracellular DNA and RNA sensing. Nat Rev Microbiol (2016) 14:360–73. 10.1038/nrmicro.2016.45 27174148 PMC5072394

[2] Kim T Pazhoor S Bao M Zhang Z Hanabuchi S Facchinetti V Aspartate-glutamate-alanine-histidine box motif (DEAH)/RNA helicase A helicases sense microbial DNA in human plasmacytoid dendritic cells.Proc Natl Acad Sci U S A (2010) 107:15181–6. 10.1073/pnas.1006539107 20696886 PMC2930588

[3] Zhang ZQ Kim T Bao MS Facchinetti V Jung SY Ghaffari AA DDX1, DDX21, and DHX36 helicases form a complex with the adaptor molecule TRIF to sense dsRNA in dendritic cells. Immunity (2011) 34:866–78. 10.1016/j.immuni.2011.03.027 21703541 PMC3652560

[4] Li J Hu L Liu Y Huang L Mu Y Cai X DDX19A senses viral RNA and mediates NLRP3-dependent inflammasome activation. J Immunol (2015) 195:5732–49. 10.4049/jimmunol.1501606 26538395

[5] Lattmann S Giri B Vaughn JP Akman SA Nagamine Y. Role of the amino terminal RHAU-specific motif in the recognition and resolution of guanine quadruplex-RNA by the DEAH-box RNA helicase RHAU. Nucleic Acids Res (2010) 38:6219–33. 10.1093/nar/gkq372 20472641 PMC2952847

[6] Kappes MA Faaberg KS. PRRSV structure, replication and recombination: origin of phenotype and genotype diversity. Virology (2015) 479:475–86. 10.1016/j.virol.2015.02.012 25759097 PMC7111637

[7] Snijder EJ Kikkert M Fang Y Arterivirus molecular biology and pathogenesis. J Gen Virol (2013) 94:2141–63. 10.1099/vir.0.056341-0 23939974

[8] Shi CX Liu YL Ding YZ Zhang YG Zhang J. PRRSV receptors and their roles in virus infection. Arch Microbiol (2015) 197:503–12. 10.1007/s00203-015-1088-1 25666932

[9] Spilman MS Welbon C Nelson E Dokland T. Cryo-electron tomography of porcine reproductive and respiratory syndrome virus: organization of the nucleocapsid. J Gen Virol (2009) 90:527–35. 10.1099/vir.0.007674-0 19218197

[10] Zhang R Chen C Sun Z Tan F Zhuang J Tian D Disulfide linkages mediating nucleocapsid protein dimerization are not required for porcine arterivirus infectivity. J Virol (2012) 86:4670–81. 10.1128/JVI.06709-11 22301142 PMC3318635

[11] Gomez-Laguna J Salguero FJ Barranco I Pallares FJ Rodriguez-Gomez IM Bernabe A Cytokine expression by macrophages in the lung of pigs infected with the porcine reproductive and respiratory syndrome virus. J Comp Pathol (2010) 142:51–60. 10.1016/j.jcpa.2009.07.004 19691969 PMC7126906

[12] Lee YJ Lee C Cytokine production in immortalized porcine alveolar macrophages infected with porcine reproductive and respiratory syndrome virus. Vet Immunol Immunopatho006C (2012) 150:213–20. 10.1016/j.vetimm.2012.09.007 23041033

[13] Barranco I Gomez-Laguna J Rodriguez-Gomez IM Salguero FJ Pallares FJ Carrasco L. Differential expression of proinflammatory cytokines in the lymphoid organs of porcine reproductive and respiratory syndrome virus-infected pigs. Transbound Emerg Dis (2012) 59:145–53. 10.1111/j.1865-1682.2011.01252.x 21848934

[14] Lunney JK Fang Y Ladinig A Chen N Li Y Rowland B Porcine reproductive and respiratory syndrome virus (PRRSV): pathogenesis and interaction with the immune system. Annu Rev Anim Biosci (2016) 4:129–54. 10.1146/annurev-animal-022114-111025 26646630

[15] Huang C Zhang Q Feng WH. Regulation and evasion of antiviral immune responses by porcine reproductive and respiratory syndrome virus. Virus Res (2015) 202:101–11. 10.1016/j.virusres.2014.12.014 25529442 PMC7132515

[16] Wang R Zhang YJ. Antagonizing interferon-mediated immune response by porcine reproductive and respiratory syndrome virus. Biomed Res Int (2014) 2014:315470. 10.1155/2014/315470 25101271 PMC4101967

[17] Xiao YQ Ma ZX Wang R Yang LP Nan YC Zhang YJ. Downregulation of protein kinase PKR activation by porcine reproductive and respiratory syndrome virus at its early stage infection. Vet Microbiol (2016) 187:1–7. 10.1016/j.vetmic.2016.03.004 27066702

[18] Du J Ge X Liu Y Jiang P Wang Z Zhang R Targeting swine leukocyte antigen class I molecules for proteasomal degradation by the nsp1alpha replicase protein of the Chinese highly pathogenic porcine reproductive and respiratory syndrome virus strain JXwn06. J Virol (2015) 90:682–93. 10.1128/JVI.02307-15 26491168 PMC4702659

[19] Subramaniam S Beura LK Kwon B Pattnaik AK Osorio FA Amino acid residues in the non-structural protein 1 of porcine reproductive and respiratory syndrome virus involved in down-regulation of TNF-alpha expression in vitro and attenuation in vivo. Virology (2012) 432:241–9. 10.1016/j.virol.2012.05.014 22699004

[20] van Gucht S van Reeth K Pensaert M Interaction between porcine reproductive-respiratory syndrome virus and bacterial endotoxin in the lungs of pigs: potentiation of cytokine production and respiratory disease. J Clin Microbiol (2003) 41:960–6. 10.1128/JCM.41.3.960-966.2003 12624016 PMC150282

[21] Jing H Fang L Wang D Ding Z Luo R Chen H Porcine reproductive and respiratory syndrome virus infection activates NOD2-RIP2 signal pathway in MARC-145 cells. Virology (2014) 45(8–459):162–71. 10.1016/j.virol.2014.04.031 24928048

[22] Bi J Song S Fang L Wang D Jing H Gao L Porcine reproductive and respiratory syndrome virus induces IL-1beta production depending on TLR4/MyD88 pathway and NLRP3 inflammasome in primary porcine alveolar macrophages. Mediators Inflamm (2014) 2014:403515 10.1155/2014/403515 24966466 PMC4055429

[23] Luo R Fang L Jiang Y Jin H Wang Y Wang D Activation of NF-kappaB by nucleocapsid protein of the porcine reproductive and respiratory syndrome virus. Virus Genes (2011) 42:76–81. 10.1007/s11262-010-0548-6 21063763

[24] Livak KJ Schmittgen TD. Analysis of relative gene expression data using real-time quantitative PCR and the 2(-delta delta C(T)) method. Methods (2001) 25:402–8. 10.1006/meth.2001.1262 11846609

[25] Yoo JS Takahasi K Ng CS Ouda R Onomoto K Yoneyama M DHX36 enhances RIG-I signaling by facilitating PKR-mediated antiviral stress granule formation. PLoS Pathog (2014) 10(3):e1004012. 10.1371/journal.ppat.1004012 24651521 PMC3961341

[26] Fu Y Quan R Zhang H Hou J Tang J Feng WH Porcine reproductive and respiratory syndrome virus induces interleukin-15 through the NF-kappaB signaling pathway. J Virol (2012) 86:7625–36. 10.1128/JVI.00177-12 22573868 PMC3416278

[27] Duan E Wang D Luo R Luo J Gao L Chen H Porcine reproductive and respiratory syndrome virus infection triggers HMGB1 release to promote inflammatory cytokine production. Virology (2014) 468-470:1–9. 10.1016/j.virol.2014.07.046 25129433

[28] Akira S Uematsu S Takeuchi O Pathogen recognition and innate immunity. Cell (2006) 124:783–801. 10.1016/j.cell.2006.02.015 16497588

[29] Yu Y Wang R Nan Y Zhang L Zhang Y. Induction of STAT1 phosphorylation at serine 727 and expression of proinflammatory cytokines by porcine reproductive and respiratory syndrome virus. PLoS One (2013) 8:e61967. 10.1371/journal.pone.0061967 23637938 PMC3634824

[30] Pineyro PE Subramaniam S Kenney SP Heffron CL Gimenez-Lirola LG Meng XJ. Modulation of proinflammatory cytokines in monocyte-derived dendritic cells by porcine reproductive and respiratory syndrome virus through interaction with the porcine intercellular-adhesion-molecule-3-grabbing nonintegrin. Viral Immunol (2016) 29(10):546–56. 10.1089/vim.2016.0104 27643915

[31] Lee SM Kleiboeker SB. Porcine arterivirus activates the NF-kappaB pathway through IkappaB degradation. Virology (2005) 342:47–59. 10.1016/j.virol.2005.07.034 16129468 PMC7111765

[32] Kurt-Jones EA Popova L Kwinn L Haynes LM Jones LP Tripp RA Pattern recognition receptors TLR4 and CD14 mediate response to respiratory syncytial virus. Nat Immunol (2000) 1:398–401. 10.1038/80833 11062499

[33] Dosch SF Mahajan SD Collins AR. SARS coronavirus spike protein-induced innate immune response occurs via activation of the NF-kappa B pathway in human monocyte macrophages in vitro. Virus Res (2009) 142:19–27. 10.1016/j.virusres.2009.01.005 19185596 PMC2699111

[34] Jourdan SS Osorio F Hiscox JA. An interactome map of the nucleocapsid protein from a highly pathogenic North American porcine reproductive and respiratory syndrome virus strain generated using SILAC-based quantitative proteomics. Proteomics (2012) 12:1015–23. 10.1002/pmic.201100469 22522808 PMC7167637

[35] Liu L Lear Z Hughes DJ Wu W Zhou EM Whitehouse A Resolution of the cellular proteome of the nucleocapsid protein from a highly pathogenic isolate of porcine reproductive and respiratory syndrome virus identifies PARP-1 as a cellular target whose interaction is critical for virus biology. Vet Microbiol (2015) 176:109–19. 10.1016/j.vetmic.2014.11.023 25614100 PMC4414928

[36] Chen Q Liu Q Liu D Wang D Chen H Xiao S Molecular cloning, functional characterization and antiviral activity of porcine DDX3X. Biochem Biophys Res Commun (2014) 443:1169–75. 10.1016/j.bbrc.2013.12.098 24380861

[37] Zhao S Ge X Wang X Liu A Guo X Zhou L The DEAD-box RNA helicase 5 positively regulates the replication of porcine reproductive and respiratory syndrome virus by interacting with viral Nsp9 in vitro. Virus Res (2015) 195:217–24. 10.1016/j.virusres.2014.10.021 25449571 PMC7114378

[38] Liu L Tian J Nan H Tian MM Li Y Xu XD Porcine reproductive and respiratory syndrome virus nucleocapsid protein interacts with Nsp9 and cellular DHX9 to regulate viral RNA synthesis. J Virol (2016) 90:5384–98. 10.1128/Jvi.03216-15 27009951 PMC4934760

[39] Booy EP McRae EK McKenna SA. Biochemical characterization of G4 quadruplex telomerase RNA unwinding by the RNA helicase RHAU. Methods Mol Biol (2015) 1259:125–35. 10.1007/978-1-4939-2214-7_9 25579584

[40] Lattmann S Stadler MB Vaughn JP Akman SA Nagamine Y. The DEAH-box RNA helicase RHAU binds an intramolecular RNA G-quadruplex in TERC and associates with telomerase holoenzyme. Nucleic Acids Res (2011) 39:9390–404. 10.1093/nar/gkr630 21846770 PMC3241650

[41] Chalupnikova K Lattmann S Selak N Iwamoto F Fujiki Y Nagamine Y. Recruitment of the RNA helicase RHAU to stress granules via a unique RNA-binding domain. J Biol Chem (2008) 283:35186–98. 10.1074/jbc.M804857200 18854321 PMC3259895

[42] Beura LK Sarkar SN Kwon B Subramaniam S Jones C Pattnaik AK Porcine reproductive and respiratory syndrome virus nonstructural protein 1beta modulates host innate immune response by antagonizing IRF3 activation. J Virol (2010) 84:1574–84. 10.1128/JVI.01326-09 19923190 PMC2812326

[43] Sagong M Lee C Porcine reproductive and respiratory syndrome virus nucleocapsid protein modulates interferon-beta production by inhibiting IRF3 activation in immortalized porcine alveolar macrophages. Arch Virol (2011) 156:2187–95. 10.1007/s00705-011-1116-7 21947566 PMC7086947

[44] Dong J Xu S Wang J Luo R Wang D Xiao S Porcine reproductive and respiratory syndrome virus 3C protease cleaves the mitochondrial antiviral signalling complex to antagonize IFN-beta expression. J Gen Virol (2015) 96:3049–58. 10.1099/jgv.0.000257 26253126 PMC5410108

[45] Han M Kim CY Rowland RR Fang Y Kim D Yoo D. Biogenesis of non-structural protein 1 (nsp1) and nsp1-mediated type I interferon modulation in arteriviruses. Virology (2014) 458-459:136–50. 10.1016/j.virol.2014.04.028 24928046

[46] Sun Z Li Y Ransburgh R Snijder EJ Fang Y. Nonstructural protein 2 of porcine reproductive and respiratory syndrome virus inhibits the antiviral function of interferon-stimulated gene 15. J Virol (2012) 86:3839–50. 10.1128/JVI.06466-11 22258253 PMC3302520

[47] Chen Z Liu S Sun W Chen L Yoo D Li F Nuclear export signal of PRRSV NSP1alpha is necessary for type I IFN inhibition. Virology (2016) 499:278–87. 10.1016/j.virol.2016.07.008 27718457

[48] Wang R Nan Y Yu Y Zhang YJ Porcine reproductive and respiratory syndrome virus Nsp1beta inhibits interferon-activated JAK/STAT signal transduction by inducing karyopherin-alpha1 degradation. J Virol (2013) 87:5219–28. 10.1128/JVI.02643-12 23449802 PMC3624296

[49] Chen Z Li M He Q Du J Zhou L Ge X The amino acid at residue 155 in nonstructural protein 4 of porcine reproductive and respiratory syndrome virus contributes to its inhibitory effect for interferon-beta transcription in vitro. Virus Res (2014) 189:226–34. 10.1016/j.virusres.2014.05.027 24911239

[50] Wootton SK Rowland RRR Yoo D. Phosphorylation of the porcine reproductive and respiratory syndrome virus nucleocapsid protein. J Virol (2002) 76:10569–76. 10.1128/Jvi.76.20.10569-10576.2002 12239338 PMC136587

[51] Kenney SP Meng XJ. An SH3 binding motif within the nucleocapsid protein of porcine reproductive and respiratory syndrome virus interacts with the host cellular signaling proteins STAMI, TXK, Fyn, Hck, and cortactin. Virus Res (2015) 204:31–9. 10.1016/j.virusres.2015.04.004 25882913

[52] Wang X Bai J Zhang L Wang X Li Y Jiang P. Poly(A)-binding protein interacts with the nucleocapsid protein of porcine reproductive and respiratory syndrome virus and participates in viral replication. Antiviral Res (2012) 96:315–23. 10.1016/j.antiviral.2012.09.004 22985629

[53] Rowland RRR Schneider P Fang Y Wootton S Yoo D Benfield DA. Peptide domains involved in the localization of the porcine reproductive and respiratory syndrome virus nucleocapsid protein to the nucleolus. Virology (2003) 316:135–45. 10.1016/S0042-6822(03)00482-3 14599798 PMC7125632

[54] Lee C Hodgins D Calvert JG Welch SKW Jolie R Yoo D. Mutations within the nuclear localization signal of the porcine reproductive and respiratory syndrome virus nucleocapsid protein attenuate virus replication. Virology (2006) 346:238–50. 10.1016/j.virol.2005.11.005 16330065 PMC7172752

[55] Lee C Calvert JG Welich SKW Yoo D. A DNA-launched reverse genetics system for porcine reproductive and respiratory syndrome virus reveals that homodimerization of the nucleocapsid protein is essential for virus infectivity. Virology (2005) 331:47–62. 10.1016/j.virol.2004.10.026 15582652

