# Supplementary material for: DExD/H-Box Helicase 36 Signaling via Myeloid Differentiation Primary Response Gene 88 Contributes to NF-κB Activation to Type 2 Porcine Reproductive and Respiratory Syndrome Virus Infection
Source: Front Immunol. 2017 Oct 23;8:1365. doi: 10.3389/fimmu.2017.01365 (PMC5662876; doi:10.3389/fimmu.2017.01365)
Supplement: Supplementary file 1 [file Table_1.DOCX]

| **DHX36**^#^  **primer** **sequence (5′-3′)** |
| --- |
| **WT** **(Forward)** TTTGAATTCATGAGCTATGACTACCATCAGAGCT  **WT** **( Reverse)** TTTCTCGAGTCAGCTATAATATCCATCCTGGAAT  **a** **(Forward)** TTTGAATTCATGAGCTATGACTACCATCAGAGCT  **a** **( Reverse)** TTTCTCGAGTCAGAGTGACACTGCTTCATTCGATGG  **b** **(Forward)** TTTGAATTCATGAGCTATGACTACCATCAGAGCT  **b** **( Reverse)** TTTCTCGAGTCAGGAAGGCAGCTTTTCCCTGAAAT  **c** **(Forward)** TTTGAATTCAGTGATAAAGATTCAGAAGCAC  **c** **( Reverse)** TTTCTCGAGTCAGAGTGACACTGCTTCATTCGATGG  **d** **(Forward)** TTTGAATTCAGTGATAAAGATTCAGAAGCAC  **d** **(Reverse)** TTTCTCGAGTCAGCTATAATATCCATCCTGGAAT  **e** **(Forward)** TTTGAATTCTATAATGGTCTTAGAGCAAGCCTTC  **e** **(Reverse)** TTTCTCGAGTCAGCTATAATATCCATCCTGGAAT |

**Table S1. The sequences of primers used for the cloning of genes.**

**(Note:# represented GenBank accession number: GACC01000505.1)**
