# Supplementary material for: DExD/H-Box Helicase 36 Signaling via Myeloid Differentiation Primary Response Gene 88 Contributes to NF-κB Activation to Type 2 Porcine Reproductive and Respiratory Syndrome Virus Infection
Source: Front Immunol. 2017 Oct 23;8:1365. doi: 10.3389/fimmu.2017.01365 (PMC5662876; doi:10.3389/fimmu.2017.01365)
Supplement: Supplementary file 2 [file Table_2.DOCX]

| **Name (Refseq)** | **Forward primer (5'-3')** | | **Reverse primer (5'-3')** | |
| --- | --- | --- | --- | --- |
| **DHX36 (XM_015131845.1)**  **MyD88 (NM_001130681.1)**  **TRIF (NM_001130428.1)**  **IL-6 (NM_001042733.2)**  **IL-8 (NM_001032965.1)**  **TNF-α (NM_001047149.1)**  **RANTES (NM_001032850.1)**  **GAPDH (NM_001195426.1)** | | AAAGGACAGTTTGCTGAGC  TCTCCTCCACATCCTCCCTT  CCCCTGCCCTGTCAAACAC  GCTGCAGGCACAGAACCA  CTGGCGGTGGCTCTCTTG  TCCTCAGCCTCTTCTCCTTCCT  TCTGCGCTCCTGCATCTG  TCATGACCACAGTCCATGCC | | GGATGAATAGCAACCAGGC  TCAAAGTCCATCTCCTCCG  GCACGGCTTGGTATTTGGA  AAAGCTGCGCAGGATGAGA  CCTTGGCAAAACTGCACCTT  ACTCCAAAGTGCAGCAGACAGA  GGGCAATGTAGGCAAAGCA  GGATGACCTTGCCCACAGCC |

**Table S2. The sequences of primers used in MARC-145 cells for real-time PCR.**
